# PHYSICAL INTIMACY IN DAILY LIVES OF OLDER ROMANTIC COUPLES: LINKS WITH MOMENTARY AFFECT AND DAILY CORTISOL LEVELS

**DOI:** 10.1093/geroni/igac059.730

**Published:** 2022-12-20

**Authors:** Karolina Kolodziejczak, Johanna Drewelies, Theresa Pauly, Nilam Ram, Christiane Hoppmann, Denis Gerstorf

**Affiliations:** Medical School Berlin, Berlin, Berlin, Germany; Humboldt University Berlin, Berlin, Berlin, Germany; University of Zurich, Zurich, Zurich, Switzerland; Stanford University, Stanford, California, United States; University of British Columbia, Vancouver, British Columbia, Canada; Humboldt Universität zu Berlin, Berlin, Berlin, Germany

## Abstract

Physical intimacy is assumed to benefit well-being through stress-buffering and mood-improving processes. Although partnered older adults often report wishing for and experiencing physical intimacy, inquiries about how intimacy is linked to affect and stress in older couples’ daily lives remain scarce. We examined self-report and salivary cortisol data from 120 German couples (Mage= 71.6, SDage= 5.94) obtained up to seven times per day over seven consecutive days. In moments when participants experienced more physical intimacy, women reported less negative affect, and men more positive affect. Experiencing more overall physical intimacy was associated with more positive affect and less negative affect in women, and lower daily cortisol in men. More overall intimacy wished was related to more negative affect in women and men, and to higher daily cortisol in men. We conclude that physical intimacy relates to indicators of well-being in older couples’ daily lives and consider routes for future inquiry.

